# Reconsidering a silent variant: *SGCA*’s role in atypical cardiomyopathy

**DOI:** 10.1038/s41431-025-01981-z

**Published:** 2025-12-04

**Authors:** Smadar Horowitz-Cederboim, Ronit Hoffman-Lipschuetz, Ronen Durst, Shoshi Shpitzen, Ayelet Shauer, Donna R. Zwas, Chaggai Rosenbluh, Israel Antman, Avital Eilat, Tamar Harel, Orr Tomer, Vardiella Meiner

**Affiliations:** 1 The Heart Institute, Hadassah Hebrew University Medical Center, Jerusalem, Israel; 2 Hadassah Center for Cardiogenetics, Hadassah Hebrew University Medical Center, Jerusalem, Israel; 3 Department of Genetics, Hadassah Hebrew University Medical Center, Jerusalem, Israel

**Keywords:** Cardiomyopathies, Genetics research, Medical genetics, Functional genomics, Consanguinity

## Abstract

Limb-girdle muscular dystrophy type R3 (LGMDR3) is caused by pathogenic *SGCA* variants and typically presents as progressive muscle weakness with limited cardiac manifestations. We investigated five consanguineous families with substantial left ventricular dysfunction, arrhythmias, and life-threatening ventricular tachyarrhythmias. Cardiac assessments, including echocardiography, Holter monitoring, and cardiac magnetic resonance imaging, revealed a spectrum of findings from mild asymptomatic dysfunction to severe dilated cardiomyopathy and malignant arrhythmia requiring implantable cardioverter-defibrillators. Initial evaluation with exome sequencing showed no conclusive results until a genotype-phenotype correlation reanalysis pinpointed a single homozygous synonymous *SGCA* variant shared by all affected individuals. Despite being distant from canonical splice sites, this variant disrupted normal mRNA splicing, leading to aberrant transcripts and a presumably nonfunctional or structurally altered α-sarcoglycan protein. This study broadens the recognized clinical spectrum of LGMDR3 by revealing significant cardiac involvement and exemplifies how a splice-disrupting synonymous variant, initially classified as likely benign, can underlie a severe cardiac phenotype and merit reclassification. Our findings highlight the importance of integrating genotype-phenotype correlation with functional studies to improve diagnostic accuracy, particularly in underrepresented populations.

## Introduction

The genetic landscape of inherited cardiomyopathies is characterized by a complex array of inheritance patterns. While autosomal dominant is most common, autosomal recessive, X-linked, and mitochondrial inheritance patterns are also recognized [1]. Recessive forms are more frequent in consanguineous populations and may include myopathic features.

Limb-girdle muscular dystrophy type R3 (LGMDR3), previously known as LGMD2D [2], is an autosomal recessive disorder caused by pathogenic variants in the *SGCA* gene and characterized by progressive proximal muscle weakness ranging from asymptomatic to severe. Unlike other muscular dystrophies, cardiac involvement in LGMDR3 is relatively uncommon. Studies suggest that overt cardiomyopathy is observed in ~10% of LGMDR3 patients [3, 4].

Here, we describe findings from five consanguineous families in which affected individuals had undergone exome testing for various indications, including cardiac symptoms with muscle involvement, isolated myopathy, or both. Initial analyses provided no conclusive results. However, subsequent reanalysis incorporating genotype-phenotype correlation within our cohort revealed a homozygous synonymous variant in *SGCA*. This variant led to an atypical form of LGMDR3 characterized by significant cardiac involvement.

This study highlights the value of periodic reanalysis in complex presentations. It also underscores the need for careful evaluation of variants initially classified as benign, given their potential to hold substantial clinical significance.

## Subjects and methods

### Ethical considerations

All participants provided written informed consent for clinical data collection and genetic studies, approved by the Hadassah Medical Center Helsinki committee (0151-20-HMO). No identifiable images or videos of participants are included in this manuscript.

### Exome sequencing

Genomic DNA was extracted from whole blood samples of the following individuals: A-II-1, A-II-3, B-III-1, C-III-1, D-III-2, E-II-1, and E-II-3. Exonic sequences from DNA were enriched with the SureSelect Human All Exon 50 Mb V5 Kit (Agilent Technologies, Santa Clara, California, USA), or with the xGen Exome Research Panel IDT-V2 combined with the xGen Human mtDNA Research Panel v1.0 (IDT, Coralville, Iowa, USA). Sequences were generated on a HiSeq2500 or NovaSeq6000 sequencing system (Illumina, San Diego, California, USA) as 125- or 150-bp paired-end runs. Read alignment and variant calling of single-nucleotide variants, structural variants, and copy-number variants were performed on the Geneyx Analysis platform [5] using Illumina DRAGEN Bio-IT. The human genome assembly hg19 (GRCh37) was used as reference. Variants were annotated on the Geneyx Analysis annotation engine. Exome analysis of the probands yielded 44-105 million reads, with a mean coverage of 63-136X.

### Segregation analysis

An amplicon containing the *SGCA* variant was amplified by conventional PCR of genomic DNA from probands and all available parents and siblings and analyzed by Sanger dideoxy nucleotide sequencing with forward and reverse primers as indicated in Table S1.

### Clinical evaluation

All individuals homozygous for the variant underwent comprehensive evaluation, including blood tests for hepatocellular enzymes and creatine phosphokinase (CPK) levels, a resting electrocardiogram (ECG), 24-h Holter monitoring with 3 leads (Lifecard, Spacelabs Healthcare, Snoqualmie, Washington, United States), two-dimensional echocardiography, and cardiac magnetic resonance (CMR) imaging. Participants were scanned on either the 1.5T Avanto Siemens (Munich, Germany) or the 3T Philips Ingenia (Amsterdam, Netherlands) scanners. Assessment of left and right ventricular (LV and RV, respectively) systolic function on CMR was determined in accordance with recent criteria for the evaluation of cardiomyopathies. Late gadolinium enhancement (LGE) imaging was performed 10–15 min after intravenous administration of 0.2 mmol/kg of gadoterate-meglumine (Dotarem, manufactured by Guerbet, Villepinte, France) via short-axis breath-hold phase-sensitive inversion recovery images scanning the entire length of the myocardium. The endocardial and epicardial borders of the LV myocardium in end-diastole and end-systole were traced manually on the short-axis slices. The percent delayed enhancement was automatically determined for myocardial enhancement at least 5 standard deviations above a sample of normal remote myocardium as defined by the reader (RD). The analysis was done on 3D Synapse software by Fujifilm (Tokyo, Japan). After completing the evaluation, each patient was evaluated in consultation by a cardiologist specializing in inherited arrhythmias.

### Cell culture and generation of cell lines

Primary fibroblasts were grown from skin-punch biopsies (patient B-III-1) and maintained in Dulbecco’s modified Eagle’s medium supplemented with 15% Fetal Calf Serum, 1% L-glutamine, and 1% penicillin-streptomycin antibiotics (Biological Industries Beit Haemek, Israel).

Primary fibroblasts were immortalized using a lentiviral vector expressing human telomerase reverse transcriptase (hTERT). The lentivirus was produced by transfecting HEK293T cells (ATCC, Manassas, Virginia, USA) with the following plasmids: pLV-UBC-hTERT (Addgene #114316), psPax2 (Addgene #12260) and pMD2.G (Addgene #12259). All plasmids were obtained from Addgene (Watertown, Massachusetts, USA). Following transduction, fibroblasts were selected using blasticidin (Bio prep, Omer, Israel) at a concentration of 10 μg/mL.

### RNA/cDNA analysis

RNA was isolated from patient (B-III-1) and control fibroblasts by TRIzol reagent extraction. cDNA was prepared from 1 μg RNA using the qScript cDNA Synthesis Kit (Quantabio). Target regions within *SGCA* were amplified by PCR reactions using VeriFi™ DNA Polymerase Mix Red (PCR Biosystems, London, UK) with forward and reverse primers as indicated in Table S1. Housekeeping gene *B-actin* served as loading control to normalize expression levels. The resultant fragments were separated by 1.5% (w/v) agarose gel electrophoresis, and their sequences were determined by Sanger sequencing.

### Genomic DNA extraction

Genomic DNA was extracted from affected and control fibroblasts using the Blood & Cell Culture DNA Mini Kit (QIAGEN, Cat. No. 13323, Germantown, USA) following the manufacturer’s protocol.

### Cycloheximide treatment and nonsense-mediated decay evaluation

Affected and control fibroblasts were seeded in 6-well plates at a density of 2 × 10⁵ cells per well. After 24 h of incubation, cells were treated with cycloheximide (CHX) at final concentrations of 0, 100, and 500 µg/ml for 24 h. Following treatment, cells were harvested, and total RNA was extracted for cDNA synthesis. PCR analysis was subsequently performed with primers provided in Table S1.

### Amplicon-based next generation sequencing

RNA samples were extracted from fibroblasts cells using the Qiagen RNA purification Kit (Qiagen, Germantown, USA). cDNA was prepared from 1 μg RNA using the qScript cDNA Synthesis Kit (Quantabio, Beverly, MA). Target cDNA regions were amplified with specific primers for the relevant region. Adapters for Illumina next-generation sequencing (NGS) were added by PCR using the IDT for Illumina UD indexes. PCR products were cleaned using KAPA pure beads (Roche Sequencing Solutions, Indianapolis, IN USA), and samples were sequenced on an Illumina NovaSeq6000 platform using 161 paired-end reads, and to a minimal depth of 1 million paired-end reads per sample. Results were visualized with the Integrative Genomics Viewer.

## Results

### Cohort description

Overall, 13 individuals are reported in this study, of whom six were female, with a mean age at presentation of 10.9 ± 7.3 years and a mean age at last follow-up of 25.8 ± 13.7 years. Clinical features are detailed in Table 1. At presentation, 8/11 had elevated CPK levels, and 3/11 showed signs of myopathy. At follow-up, all tested individuals (11/11) had elevated CPK, with ALT and AST elevated 2–3 × the upper limit of normal. Progressive weakness or myalgia was reported in 6/11, and recurrent rhabdomyolysis in 5/11. Among 12 evaluated subjects, 6 (50%) showed reduced LV systolic function, ranging from mild to severe. CMR revealed LGE in all five tested individuals, primarily in the lateral (5/5) and inferior (4/5) walls, localized to the subepicardial and mid-myocardial layers (Fig. S1B, C). Two had LV dilation; the other four were classified as non-dilated cardiomyopathy. ECG abnormalities were seen in 3/6, including low limb-lead voltage and incomplete right bundle branch block.

**Table 1:** Represents clinical abnormalities among all known/highly suspected carriers.

Family	Family A				Family B				Family C	Family D		Family E		Total
Individual	II-1	II-3	II-7	III-4	III-1	III-2	III-3	III-6	III-1	III-1	III-2	II-1	II-3	
Gender	M	M	F	M	M	F	F	M	M	F	M	F	F	
Current Age	31	28	17	3	28	30	26	18	45	13	10	46	40	
Age at first presentation	5	10	5	3	12	19	NA	NA	17	9	6	27	7	
Genotype	HOM	HOM	HOM	NA^a^	HOM	HOM	HOM	NA^a^	HOM	HOM	HOM	HOM	HOM	
Muscle weakness	–	–	+	–	+	–	NA	NA	+	+	–	+	+	55% (6/11)
Elevated CPK	+	+	+	+	+	+	NA	NA	+	+	+	+	+	100% (11/11)
Elevated liver enzymes Δ	+	+	+	+	+	+	NA	NA	+	+	+	+	+	100% (11/11)
Myoglobinuria	+	+	+	+	–	+	NA	NA	–	–	–	–	–	45% (5/11)
Cardiomyopathy (LVEF)†	38%	40%	–	–	19%	45%	–	NA	49%	–	–	–	49%	50% (6/12)
RV dysfunction	–	–	–	–	–	–	–	NA	–	–	–	–	–	0% (0/12)
Dilated LV □	115	–	–	–	189	–	–	NA	–	–	–	–	–	17% (2/12)
Abnormal ECG ‡	+	+	NA	NA	+	–	NA	NA	–	NA	NA	–	NA	50% (3/6)
LGE on CMR	+	+	NA	NA	+	+	NA	NA	+	NA	NA	NA	NA	100% (5/5)
PVCs (24 h) §	NSVT	NSVT	NA	NA	19,000	460	NA	NA	580	NA	NA	–	–	43% (3/7)
SCD / MVA	VT/VF	–	–	–	VF	–	–	SCD	–	–	–	–	–	23% (3/13)

*NA* not available data, *HOM* homozygotic carrier, *CPK* creatine-phosphokinase, *LVEF* left ventricle ejection fraction, *LV* left ventricle, *RV* right ventricle, *LGE* late gadolinium enhancement, *CMR* cardiac magnetic-resonance, *PVC* premature ventricular contractions, *SCD* sudden cardiac death, *MVA* major ventricular arrhythmia, *VT* ventricular tachycardia, *VF* ventricular fibrillation. *NSVT* episodes of non-sustained VT.

^a^Genetic results unknown (but with high level of suspicion).

Δ – elevated liver enzymes considered when alanine aminotransferase (ALT) or aspartate transaminase (AST) are at greater levels than twice their upper normal limit.

† - percentage represents LVEF, “-“ indicates normal systolic LV function.

□ - left ventricular dilatation as measured on CMR, if abnormal LVEDV/BSA (ml/m^2^) is indicated.

‡ - abnormal ECG include low voltage in limb leads, conduction disturbance, early repolarization or lateral T waves inversion.

§ - number represents PVC count per 24 h Holter results, considered abnormal if PVCs>1000/24 h or NSVTs are present.

Two individuals experienced recurrent polymorphic ventricular tachycardia (VT) or ventricular fibrillation (VF) requiring implantable cardioverter-defibrillator (ICD) implantation. Another had a brief run of monomorphic non-sustained VT (NSVT) on Holter, and two showed premature ventricular contractions (PVCs) < 1000/24 h. In family B, individual III-6 died suddenly at age 18 from unknown causes; DNA was unavailable for testing. No other rare variants were identified in individuals with severe cardiac disease, and no consistent comorbidities were observed. Notably, heterozygous carriers did not exhibit any cardiac clinical signs or symptoms to date, except for one with elevated CPK (see Table S2 for details).

### Representative clinical cases

We present three representative cases, all males aged 24–28 years at their first major clinical cardiac presentation.

#### Patient A-II-1

A 24-year-old male presented with sudden palpitations, chest pain, and weakness during physical activity. His history included elevated CPK levels and episodes of rhabdomyolysis since age 5, shared by siblings A-II-3 and A-II-7. Echocardiogram suggested non-ischemic dilated cardiomyopathy with ejection fraction (EF) of 38%. CMR showed left ventricular dysfunction (LVEF 47%) and dilatation with end-diastolic volume index (LVEDVi) of 111 mL/m^2^, normal RV function (RVEF 53%) and size (RVEDVi 89 ml/m^2^), no increased T2 signal but presence of LGE in the lateral wall at the subepicardial and mid-myocardial layers. He underwent an ICD placement due to recurrent polymorphic VT. Despite medical therapy, he had recurrent appropriate shocks, requiring multiple ablations, including epicardial. Arrhythmias have since been well controlled for 4 years.

#### Patient A-II-3

A 28-year-old male, the brother of A-II-1, presented with myocarditis-like episodes at ages 24 and 28. Both included atypical chest pain, mildly elevated troponin (0.04 ng/mL; reference <0.03 ng/mL), and marked CPK elevation (up to 15,000 μ/L). His prior history included persistent CPK elevation from age 10; he was also identified as a hepatitis C carrier. Cardiac function was normal at age 24, but at 28 he showed biventricular dysfunction (LVEF 40%, RVEF 42%). CMR revealed LGE in the lateral and inferior walls, with resolution of edema (T2 signal) and no LGE progression. He has not experienced major ventricular arrhythmias, though a 5-beat run of monomorphic NSVT was recorded on Holter. An internal loop recorder was implanted due to ongoing palpitations, with no notable events. ACE inhibitors and beta-blockers led to improved function (LVEF 50%) over several months.

#### Patient B-III-1

A 28-year-old male was successfully resuscitated following VF during physical activity. His medical history included elevated creatine kinase (CPK) levels and cardiomyopathy diagnosed 10 years earlier, with a left ventricular ejection fraction (LVEF) reported as 45% at that time. He did not receive pharmacologic treatment at that time. Echocardiography revealed severely reduced systolic function (LVEF < 20%) and left ventricular dilatation. CMR demonstrated mid-myocardial LGE in the basal inferior and lateral walls. Holter monitoring showed frequent parasystolic PVCs (23% of total beats), originating from the left anterior fascicle (Fig. S1A), refractory to antiarrhythmics. He received an ICD and later underwent PVC ablation, with limited improvement in systolic function (LVEF 25%).

### Exome sequencing reveals a shared homozygous variant in SGCA

**Fig. 1:**
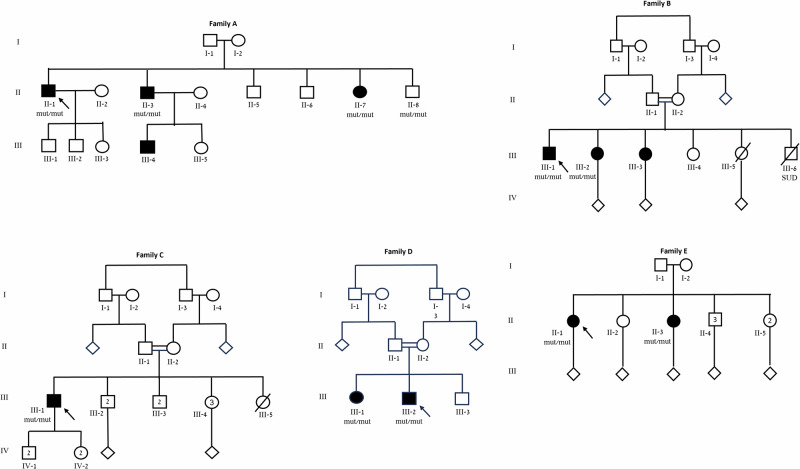
Pedigrees of five families harboring the *SGCA* variant. Squares represent males, circles females. Filled symbols indicate individuals presenting with the disease phenotype caused by the *SGCA* variant described in this study. Diamonds represent multiple siblings. Slashed symbols denote deceased individuals. Arrows indicate probands. Clear consanguinity is evident in families B, C, and D, whereas in families A and E the degree of consanguinity is not clear.

Whole-exome sequencing was undertaken on 7 individuals from 5 families (Fig. 1). Initial analysis was uninformative. Subsequent reanalysis identified a homozygous synonymous variant in *SGCA* (MIM: 600119), designated as NM_000023.4:c.600G>A; NP_003997.1:p.Val200=; NC_000017.11:g.50169107G>A (GRCh38). All families were of Arab Muslim origin and originated from the same village, characterized by high endogamy. Clear consanguinity was evident in families B, C, and D, whereas in families A and E it was not formally documented. However, both the parents of the probands and, in family A, the probands themselves are married within the same village, consistent with the high endogamy and shared ancestry of this community. Analysis of runs of homozygosity (ROH) was performed using exome data from five affected individuals. The two brothers in family A shared a homozygous region of 11.6 Mb around the SGCA variant. The other three individuals shared a separate 14.4 Mb homozygous segment, with only 0.65 Mb of overlap across all five individuals. Notably, the SGCA variant was located near the boundary of both segments, consistent with a shared ancestral haplotype and suggesting a founder effect. The allele frequency was 0.07% in our dataset, and 5.1% within the specific village. Following identification of this variant, three additional families with overlapping phenotypes were found to harbor the same variant.

The *SGCA* variant was initially overlooked due to its synonymous nature and distance (15 bp) from the nearest exon–intron boundary. However, the recurrence of the variant within the database and the overlapping phenotype between the cases led to further evaluation. We submitted the variant to ClinVar (SCV005888590.1; VCV000390366.9) to support future interpretation.

### SGCA variant leads to aberrant splicing

**Fig. 2:**
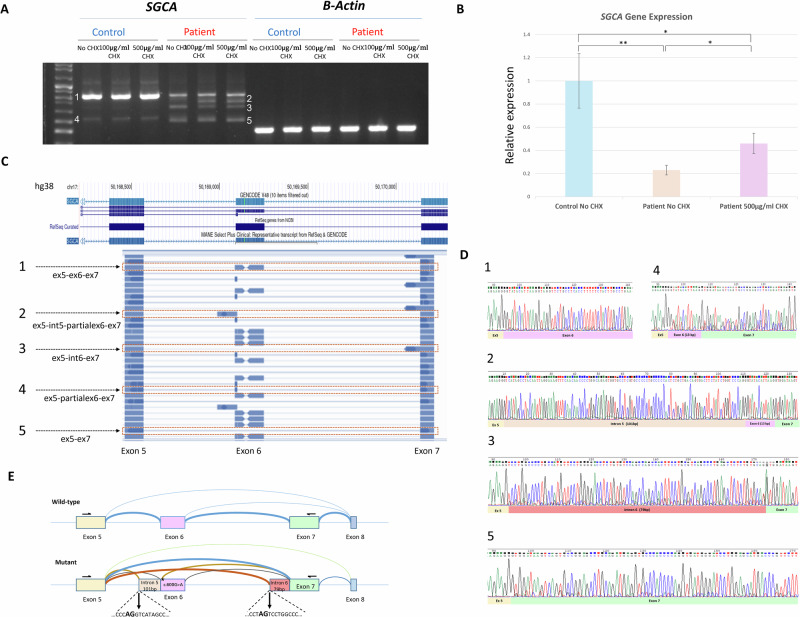
cDNA analysis of SGCA variant indicates aberrant splicing. **A** Gel electrophoresis of cDNA from patient and control fibroblasts demonstrating reduced SGCA expression caused by NMD, which is partially rescued by CHX inhibition of NMD. Aberrant splicing is demonstrated by multiple bands seen only in the affected individual. B-actin was included as a housekeeping gene. **B** Quantification of SGCA expression levels by quantitative real-time PCR demonstrates decreased expression in affected fibroblasts compared to control and partial rescue following 500 µg/ml CHX treatment. Values are presented as normalized mean ± SEM, ^*^p < 0.05, ^**^p < 0.01. **C** Amplicon-based next-generation sequencing reads in IGV viewer. **D** The five bands were excised and sequenced; representative Sanger traces from affected and control are shown. **E** Diagram of the exon skipping and intronic inclusions, indicating cryptic splice sites that were activated.

The SpliceAI algorithm [6] predicted a moderate splice-altering effect (score: 0.31). To establish the effect of the variant at the RNA level, we analyzed the cDNA sequence derived from fibroblasts of an affected individual and healthy control. Reverse transcription polymerase chain reaction (RT-PCR) analysis revealed several amplicons in the affected individual that were absent in the control sample, as demonstrated by gel electrophoresis (Fig. 2A, left panel). Treatment of affected fibroblasts with CHX to inhibit nonsense-mediated decay (NMD) resulted in a distinct increase in the intensity of a specific band (labelled band 2 in Fig. 2A, right panel). Accordingly, quantitative real-time PCR analysis demonstrated reduced levels of total *SGCA* transcripts in untreated affected fibroblasts compared to controls, while CHX treatment partially restored transcript levels (Fig. 2B). Amplicon-based NGS (AmpliSeq, Fig. 2C) and Sanger sequencing (Fig. 2D) allowed for characterization of the abnormally spliced transcripts: band 2 contained an intronic retention of 101 base pairs (bp) from intron 5 followed by 13 bp of exon 6, leading to a frameshift and premature stop codon (Fig. S2), triggering NMD; band 3 contained 79 bp from intron 6 adjacent to exon 7, and band 5 had complete skipping of exon 6 (163 bp, out of frame). A schematic representation of the observed splicing events is shown in Fig. 2E and a summary is provided in Table S3. The *SGCA* variant was predicted to abolish an exonic splicing enhancer (ESE) motif (Fig. S3) recognized by the serine/arginine-rich (SR) splicing factor, SRSF2 (also known as SC35) [7, 8]. Presumably, loss of the ESE led to skipping of exon 6 and recognition of an alternative splice acceptor site within intron 6. To ensure that there was no other intronic sequence variant in intron 6 that may lead to the mis-splicing, we sequenced intron 6 by the Sanger method. No other variant was identified, supporting the pathogenicity of the synonymous c.600G>A (*p*.Val200 = ) variant.

## Discussion

### Expanded phenotype of LGMDR3 with cardiac involvement

This study establishes a connection between a synonymous SGCA variant and an expanded LGMDR3 phenotype with significant cardiac involvement. While *SGCA* variants are typically linked to proximal muscle weakness and elevated CPK, cardiac manifestations are considered rare. All tested individuals in this cohort showed elevated liver enzymes, consistent with prior reports in other LGMD subtypes [9, 10].

Half of the evaluated individuals (6/12) demonstrated left ventricular dysfunction ranging from mild to severe. Additionally, several individuals experienced life-threatening ventricular arrhythmias, including VT/VF in two individuals, NSVT in one, and frequent PVCs in two others. Tragically, one family member experienced sudden cardiac death (SCD), though his genotype remains unknown.

This level of cardiac involvement is notable compared to prior reports of LGMDR3, in which cardiomyopathy has been primarily associated with variants affecting exon 3 [4].

A comparative study indicated that cardiomyopathy and cardiac events are less frequent in LGMDR3 than in LGMDR5, with only 12% of LGMDR3 patients exhibiting left ventricular dysfunction. The mean LVEF in LGMDR3 patients was reported as 63%, although an LVEF below 55% was correlated with increased mortality [11].

It is important to highlight, however, that the existing literature on cardiac involvement in LGMDR3 is based on small cohort studies, emphasizing the need for more extensive research in this area. In LGMDs (dominant and recessive), LGE is a common CMR finding in patients with LV dysfunction. LGE typically affects less than 10% of LV mass, predominantly appearing in the subepicardial layer or as patchy infiltrates in the mid-myocardial layer, particularly in the lateral wall [12]. Unlike other cardiomyopathies, the prognostic significance of LGE in LGMD remains undefined.

### Molecular mechanisms and functional impact of the SGCA variant

To better understand the molecular basis of these findings, it is essential to examine the role of *SGCA*. This gene encodes α-sarcoglycan, a key component of the sarcoglycan complex within the larger dystrophin-associated protein complex (DAPC) in skeletal and cardiac muscles. The DAPC plays an essential role in preserving sarcolemmal integrity during the cycles of muscle contraction and relaxation. Pathogenic *SGCA* variants include missense, nonsense, frameshift, and splice site mutations [3, 4]. Synonymous variants have long been recognized as potentially disruptive to mRNA splicing, through effects on exonic splicing regulatory elements [13–15]. In our case, the synonymous c.600G>A (*p*.Val200=) variant caused significant alterations in mRNA splicing, despite neither altering the amino acid sequence nor being positioned near a canonical splice site. RNA/cDNA analysis demonstrated that this variant, located in exon 6 (of 10), likely disrupts an ESE domain, which is critical for spliceosome positioning [16]. The variant is predicted to abolish the ESE motif recognized by the SR splicing factor SRSF2/SC35, thereby promoting exon 6 skipping during the splicing process. This disruption results in three primary aberrant transcripts: one that, as expected, skips exon 6 *(5-7-8)*, a second that incorporating 101 nucleotides from intron 5 adjacent to 13 nucleotides of exon 6 (*5-intron5partial-exon6partial-7*), and a third that skips exon 6 while incorporating 79 nucleotides from intron 6 adjacent to exon 7 *(5-intron6partial-7-8)*. The exon-skipping transcript and the 101 intronic retention transcript introduce a frameshift, potentially leading to a truncated or nonfunctional protein. Interestingly, the inclusion of 79 intronic nucleotides compensates for the frameshift caused by exon 6 skipping, preserving the reading frame (Table S3). However, this transcript remains abnormal, as it lacks exon 6 and includes an intronic sequence. The functional consequences of this altered mRNA structure are yet to be determined and warrant further investigation.

### Clinical management and implications for risk stratification

These findings carry significant implications for clinical management. The 2022 HRS guidelines recommend ICDs for primary prevention in LGMD2 patients with LVEF < 35% [17]. However, based on the arrhythmic history observed in the families studied here, shared decision-making discussions were conducted with patients exhibiting any degree of LV dysfunction and LGE on CMR.

Our internal database indicates an allele frequency of 5.1% for this *SGCA* variant within the village population. Due to the high rates of consanguinity, accurately estimating the carrier rate remains challenging. To address this significant health risk, we propose the implementation of a comprehensive population screening strategy. Such an approach aims to identify at-risk individuals early, thereby enabling preventive care and management strategies for affected families. This is illustrated by patient A-II-3, whose LV function improved following targeted therapy.

### Reevaluating variant classification

This study underscores the importance of reevaluating existing variant classifications especially in endogamous populations that are under-represented in genetic databases, where rare variants are more likely to remain uncertain [18]. The overlooked SGCA variant illustrates the complexity of interpretation and the value of iterative, expert-driven analysis. Reevaluating variant classifications considering new clinical evidence can lead to diagnoses that are more accurate and improved patient care.

This case shows the limits of relying on database annotations alone, as the variant was previously labeled likely benign in ClinVar [19]. While resources like ClinVar provide valuable platforms for unifying knowledge across laboratories, clinical geneticists should prioritize thorough analysis of the specific family under investigation. By extracting maximum information from internal data before consulting external sources, a more context-specific and comprehensive interpretation of genetic variants may be achieved. Importantly, the global description of this variant in ClinVar underscores its broader significance beyond our studied population, reinforcing the need for continuous reevaluation of genetic variants considering emerging clinical and functional data across diverse populations. In our case, the presence of the same variant in several patients from a consanguineous village, all with cardiac or muscular disease, was a key diagnostic clue likely missed without access to an internal cohort, as shown in recent work by members of our author group [20].

## Limitations and future directions

This study has several important limitations. Firstly, RNA expression analysis was conducted in fibroblasts, a suboptimal tissue choice for this condition, due to lack of expression in blood. Ideally, cardiac and skeletal muscle samples would provide better data for the observed phenotypes. Secondly, our study is limited by a small sample size drawn from a single village, potentially affecting the generalizability of our findings. Moreover, while index cases underwent comprehensive assessments, the overall small cohort size may introduce ascertainment bias in our clinical estimations.

These limitations point to several avenues for future research. Examining the differential expression of *SGCA* in relevant tissues, particularly comparing cardiac and skeletal muscle samples in males and females, would provide valuable insights into the tissue-specific effects of this variant. Exploring potential therapeutic approaches, including gene therapy and base-editing techniques, could pave the way for targeted treatments. Conducting long-term follow-up studies on affected individuals homozygous for this variant would better elucidate the natural history of this variant. Finally, investigating the prevalence and impact of this variant in other populations could reveal its broader significance.

## Conclusions

We identified a homozygous synonymous variant in SGCA (c.600G>A; *p*.Val200=) associated with an atypical presentation of LGMDR3, characterized by cardiomyopathy and significant arrhythmia. This finding broadens the phenotypic spectrum of SGCA-related disease by revealing marked cardiac involvement and illustrates how variants previously considered benign may, in fact, have substantial clinical impact.

Our study underscores several key aspects of modern genetic diagnostics: the importance of comprehensive variant analysis in consanguineous populations, the value of population-specific internal databases, and the need for cautious reinterpretation of existing variant classifications. By integrating genomic reanalysis with clinical insight, this work refines variant classification and highlights the diagnostic value of revisiting seemingly silent changes. Recognizing their pathogenic potential may ultimately enhance genetic screening and enable more precise, personalized care in inherited cardiomyopathies.

## Supplementary information

supplementary tables and figures

## Data Availability

The data that support the findings of this study are available from the corresponding author upon reasonable request.
