# Supplementary material for: Reconsidering a silent variant: SGCA’s role in atypical cardiomyopathy
Source: Eur J Hum Genet. 2025 Dec 4;34(4):528–34. doi: 10.1038/s41431-025-01981-z (PMC13046803; doi:10.1038/s41431-025-01981-z)
Supplement: Supplementary file 1 — supplementary tables and figures [file 41431_2025_1981_MOESM1_ESM.pdf]

Supplementary Material

Reconsidering a Silent Variant:

SGCA's Role in Atypical Cardiomyopathy

Smadar Horowitz-Cederboim, Ronit Hoffman-Lipschuetz, Ronen Durst, Shoshi Shpitzen, Ayelet Shauer, Donna R. Zwas, Chaggai Rosenbluh, Israel Antman, Avital Eilat, Tamar Harel, Orr Tomer, Vardiella Meiner

Hadassah Hebrew University Medical Center

Jerusalem, Israel

Table S1. Primers for Sanger sequencing, RT PCR and quantitative real-time PCR.

Gene	Forward Primer	Reverse Primer
SGCA (RNA level)	CGCTTCCTCTCAGCCTTGGGG	AGAAGAACGGGTCATGCTCC
SGCA (RNA level)	AAGTTTCAACAACCCCTGGC	TCACATTGCACCAGTCAACG
SGCA (RNA level)	GAAAAGAAGGGTCATAGCCT	AGAAGAACGGGTCATGCTCC
SGCA (DNA level)	ATTGACCAACAGAGCAGGGA	CAGAAGAACGGGTCATGCTC
SGCA (qPCR)	CAGGTCATTGAGGTCACAGC	AGGAACTCGGCTTGGTATGG
B-Actin	GCAGCTCACCATGGATGATG	AGGATGCCTCTCTTGCTCTG
RPLPO	GAAACTCTGCATTCTCGCTTCC	GACTCGTTTGTACCCGTTGATG

Table S2. Clinical and laboratory findings among heterozygous SGCA c.600G>A (p.Val200=) carriers.

**Family**	**Family A**			**Family B**			**Total**
Individual	I-1	I-2	II-6	II-1	II-2	III-4	
Gender	M	F	M	M	F	F	
Current Age	55	52	26	56	51	26	
Genotype	HTZ	HTZ	HTZ	HTZ	HTZ	HTZ	
Muscle weakness	-	-	-	-	-	-	**0% (0/6)**
Elevated CPK	+	-	NA	NA	-	-	**25% (1/4)**
Elevated liver enzymes	-	-	NA	-	-	-	**0% (0/5)**
Myoglobinuria	-	-	-	-	-	-	**0% (0/6)**
Cardiomyopathy (LVEF)	-	-	-	NA	NA	-	**0% (0/4)**
Dilated LV	-	-	-	NA	NA	-	**0% (0/4)**
Abnormal ECG	-	-	NA	-	-	-	**0% (0/5)**
PVCs (24hrs)	-	-	NA	NA	NA	-	**0% (0/3)**
SCD / MVA	-	-	-	-	-	-	**0% (0/6)**

HTZ - heterozygous; CPK - creatine phosphokinase; LV - left ventricle; LVEF - left ventricular ejection fraction; PVCs - premature ventricular contractions; VT/VF - ventricular tachycardia/fibrillation; NA - not available.The table summarizes cardiac and biochemical test results of six heterozygous carriers across two families.All individuals were asymptomatic. One carrier (A-I-1) showed mild CPK elevation, whereas all others demonstrated normal findings.

Table S3. Predicted reading frame and premature stop codon occurrence in *SGCA* transcripts.

Band Number	Out/In Frame	Premature Stop Codon	Detected in Affected/Control
2	Out of frame	+	Affected
3	In frame	-	Affected
4	In frame	-	Affected+ Control
5	Out of frame	-	Affected

Figure S1: Clinical imaging findings.

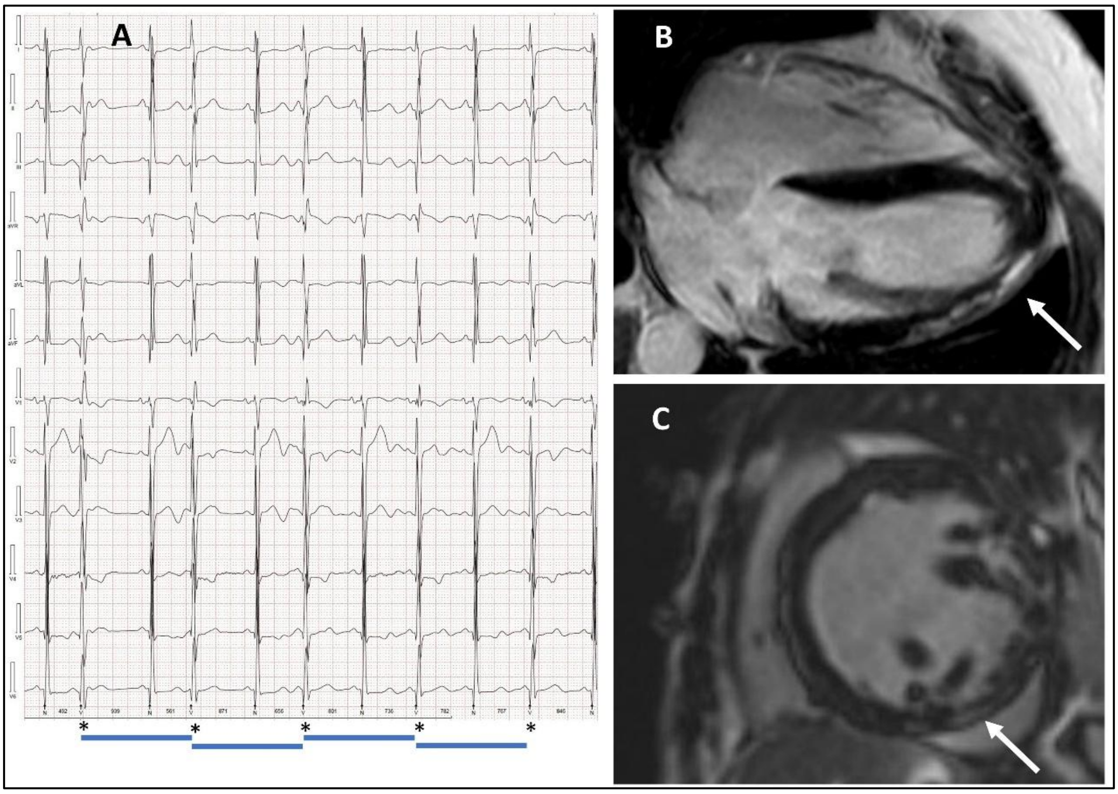

(A) Holter recording of subject B-III-1 showing parasystoles originating from the left anterior fascicle. Note the constant intervals between premature beats and varying coupling intervals with sinus beats.

(B) CMR of subject A-II-3 demonstrating LGE in the inferior wall (white arrow).

(C) CMR of subject B-III-1 also showing LGE in the inferior wall (white arrow).

Figure S2. Sanger sequencing of aberrant transcript demonstrating intron 5 retention.

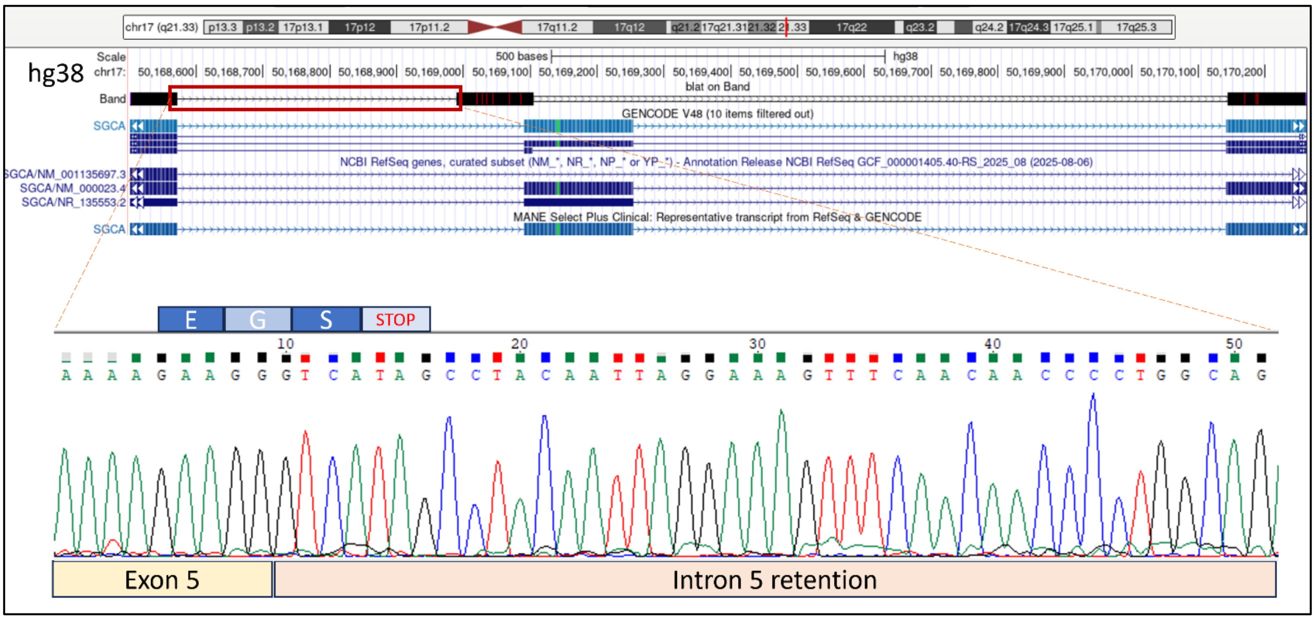

The transcript, corresponding to band 2 in the main figure, retains 101 base pairs of intron 5, resulting in a frameshift and a premature stop codon as indicated.

Figure S3: Loss of an ESE motif by SGCA c.600G>A.

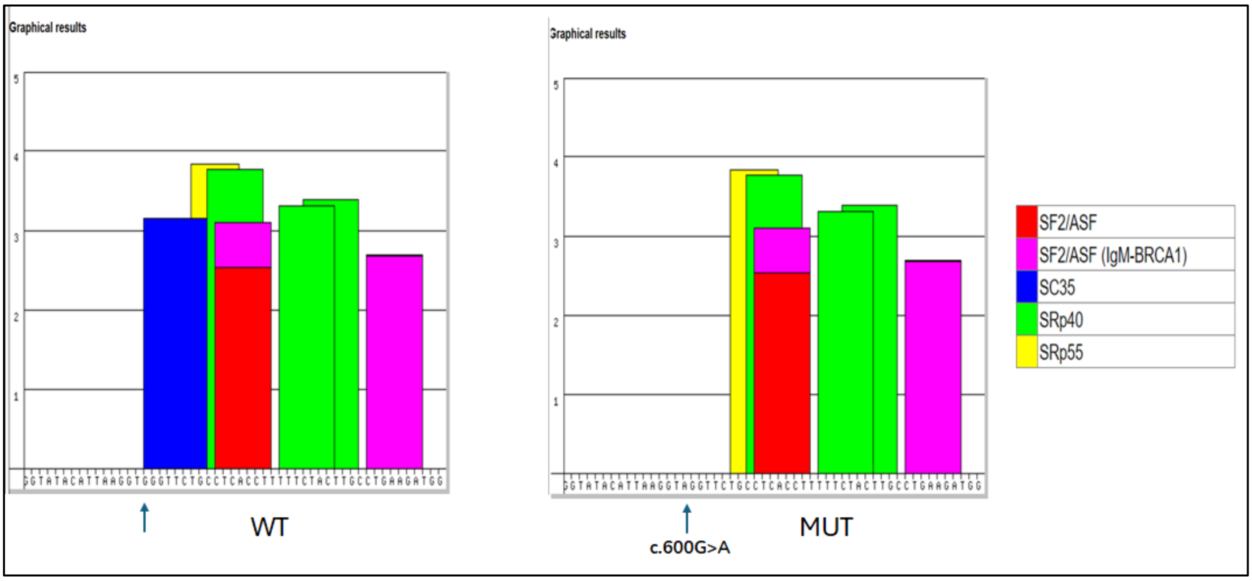

The NM_000023.4:c.600G>A (NP_003997.1:p.Val200=) variant abolishes an exonic splicing enhancer (ESE) motif recognized by the SRSF2/SC35 splicing factor, according to ESE Finder predictions. (https://esefinder.ahc.umn.edu/cgi-bin/tools/ESE3/esefinder.cgi).
